# High immune cytolytic activity in tumor‐free tongue tissue confers better prognosis in patients with squamous cell carcinoma of the oral tongue

**DOI:** 10.1002/cjp2.138

**Published:** 2019-07-19

**Authors:** Xiaolian Gu, Linda Boldrup, Philip J Coates, Robin Fahraeus, Lixiao Wang, Torben Wilms, Lena Norberg‐Spaak, Nicola Sgaramella, Karin Nylander

**Affiliations:** ^1^ Department of Medical Biosciences/Pathology Umeå University Umeå Sweden; ^2^ Regional Centre for Applied Molecular Oncology (RECAMO), Masaryk Memorial Cancer Institute Brno Czech Republic; ^3^ Institute of Molecular Genetics University Paris 7, St. Louis Hospital Paris France; ^4^ Department of Clinical Sciences/ENT Umeå University Umeå Sweden

**Keywords:** cytolytic activity, squamous cell carcinoma, oral tongue, prognosis

## Abstract

Immune cells and cytolytic activity within the tumor microenvironment are being intensively studied. Through transcriptome profiling, immune cell enumeration using the xCell tool and cytolytic activity quantification according to granzyme A (*GZMA*) and perforin (*PRF1*) mRNA levels, we investigated immunoreactivity in tumor and/or tumor‐free tongue tissue samples from 31 patients with squamous cell carcinoma of the oral tongue and 14 healthy individuals (control tongue tissues). We found significantly altered immune cell compositions (*p* < 0.001) and elevated cytolytic activity (*p* < 0.001) in tumor compared to tumor‐free samples, and altered infiltration of a subset of immune cells (e.g. CD8^+^ T cells, *p* < 0.01) as well as increased cytolytic activity (*p* < 0.001) in tumor‐free compared to control samples. Controlling for patient age at diagnosis and tumor stage, Cox regression analysis showed that high cytolytic activity in tumor‐free samples associated with improved disease‐free survival (hazard ratio= 4.20, 95% CI = 1.09–16.20, *p* = 0.037). However, the degree of cytolytic activity in tumor samples did not provide prognostic information. Taken together, our results show the presence of cancer‐related immune responses in clinically tumor‐free tongue in patients with squamous cell carcinoma of the oral tongue. Measuring cytolytic activity in tumor‐free tongue samples contralateral to tumor might thus be an effective approach to predict clinical outcome.

## Introduction

The cancer immune microenvironment has been intensively studied in the past few decades, paving the way for the recent clinical application of immunotherapies targeting immune checkpoints such as cytotoxic T‐lymphocyte associated protein 4 (CTLA4), programmed cell death 1 (PDCD1/PD1), and programmed cell death 1 ligand 1 (CD274/PDL1) 1, 2. Various immunogenomic approaches have been applied to dissect tumor‐immune cell interactions 3, 4 and accumulating evidence supports the impact of host immunity on cancer progression and response to immunotherapy 4, 5, 6, 7, 8. The latest report from the international ImmuneScore project showed that the ImmuneScore, which is derived from a digital immunohistochemistry measure of CD3^+^ and CD8^+^ lymphocytes in the tumor core and invasive margin, is a reliable prognostic biomarker in colon cancer 8. Based on transcriptome data of bulk tissue samples, a number of computational tools attempting to enumerate infiltrating immune cells are emerging 9. Recently, a novel gene signature‐based method called xCell was developed, identifying 64 immune and stromal cell types 10. By integrating the advantages of gene set enrichment with deconvolution, xCell provides a comprehensive perspective on the cellular heterogeneity of tissues 10, 11.

In this complex cellular society, cytotoxic T cells (Tc) and NK cells are two main effector cell types that can attack tumor cells directly 12. Upon exposure to transformed cells, they release perforin (a pore‐forming protein) and granzymes (a family of serine proteases) that will ultimately lead to target cell death 12, 13. Thus, local immune cytolytic activity can be quantified based on the transcript levels of perforin (*PRF1*) and granzyme A (*GZMA*) 14. Using this method, it was reported that cytolytic activity varied substantially across cancer types, with higher cytolytic activity in tumor samples from kidney, stomach, head and neck, melanoma, ovary and glioma compared to the corresponding normal tissue samples. In contrast, cytolytic activity was lower in lung cancer and colorectal cancer samples than in the corresponding normal tissues 14.

Squamous cell carcinoma of the oral tongue (SCCOT) is a subtype of squamous cell carcinoma of the head and neck (SCCHN) 15, 16. A high degree of tumor‐infiltrating lymphocytes and macrophages has been identified in SCCHN and infiltration of CD8^+^ T cells associates with good prognosis, whereas myeloid‐derived suppressor cells (MDSCs) and regulatory T cells (Tregs) associate with poor prognosis 4, 5. SCCHN represents a heterogeneous group of tumors arising from the squamous epithelium of the oral cavity, oropharynx, larynx, and hypopharynx. Despite being grouped as a single cancer type, distinct clinical, biological features and response to treatment have been seen between tumors from different subsites 16, 17, 18, 19. To characterize the immune microenvironment in SCCOT, the most common SCCHN subtype, we used transcriptome data analysis to estimate immune cell fractions and evaluated cytolytic activity according to mRNA levels of *GZMA* and *PFR1*. We show altered immune infiltration and increased cytolytic activity in tumor samples and in clinically tumor‐free tongue samples from patients with SCCOT compared to normal tongue from healthy individuals. Most importantly, we found that measures of cytolytic activity in tumor‐free samples confer prognostic information, whereas the same analysis of tumor samples does not.

## Materials and methods

### Patient material and ethical approval

This is a retrospective study of 31 patients with SCCOT. Tumor and tumor‐free samples (biopsies of clinically normal tongue tissue from the opposite side of the tongue) were collected from 21 patients. Only tumor tissue was available from eight patients, and from the remaining two patients only tumor‐free tissue could be collected for gene expression analysis. All tumor and tumor‐free samples were taken at the same time as the diagnostic biopsies, before treatment of the patients. Based on a standardized treatment protocol, when all examinations are ready, tumors are discussed at a multidisciplinary conference with participants from ENT, Oncology, Pathology, Radiology and Plastic Surgery, where treatment decisions are made. This conference should be within 18 days from arrival of the referral and therapy should start no longer than 12 days if surgical and 20 days if oncological after the conference. Patient characteristics are shown in Table 1. Tissue biopsies had been consecutively collected and some patients were included in our previous studies with different objectives 20, 21, 22, 23, 24. Biopsies taken from the lateral border of the tongue from 14 healthy volunteers not exposed to classic oral cancer risk factors (smoking and alcohol) had also been collected previously 20. The size of tumor biopsies for mRNA analysis varied between patients, with a minimum of around 3 mm. The histology of the tumor samples was described on the adjacent diagnostic biopsies taken at the same time. All histopathological analyses have been performed by the same author (KN) who as an oral pathologist also does the clinical diagnostics on these cases. Due to the limited size of the tumor‐free and healthy control samples (3–4 mm) these were only judged clinically and no histological assessment was performed. The study was approved by the Regional Ethics Review Board, Umeå, Sweden (Dnr 03‐201 and Dnr 08‐003 M) and performed in accordance with the Declaration of Helsinki. Written informed consent was obtained from all patients and healthy individuals.

**Table 1:** Clinicopathological data on patients with SCCOT

No.	ID	Age	Sex	Sample*	Localization†	TNM (clinical, 7th edition)	Stage	Treatment
1	p40	80	Female	1	3	T4N2bM0	4	RT
2	p42	68	Female	1	1	T2N0M0	2	RT, OP
3	p14	77	Female	2	2	T2N1M0	2	RT, OP
4	p24	64	Male	2	1	T1N0M0	1	OP
5	p29	64	Female	2	2	T2N0M0	2	RT
6	p68	62	Male	2	1	T2N0M0	2	OP, RT
7	p70	71	Male	2	2	T1N0M0	1	OP, RT
8	p82	19	Female	2	2	T4N0M0	4	RT, OP
9	p83	64	Female	2	2	T1N0M0	1	OP
10	p92	63	Female	2	2	T2N0M0	2	RT, OP, CYT
11	p11	78	Male	3	2	T2N0M0	2	RT, OP
12	p35	24	Female	3	1	T2N0M0	2	RT, OP
13	p49	52	Female	3	3	T4N2cM0	4	RT
14	p51	74	Male	3	1	T2N0M0	2	RT, OP
15	p56	40	Female	3	3	T2N2bM0	3	RT, OP
16	p58	61	Male	3	1	T1N0M0	1	OP
17	p59	68	Female	3	1	T2N0M0	2	RT, OP
18	p61	69	Male	3	3	T4aN0M0	4	RT
19	p65	81	Female	3	3	T2N0M0	2	OP, RT
20	p73	80	Male	3	3	T4aN0M0	4	RT
21	p76	58	Male	3	3	T4aN0M0	4	RT
22	p79	60	Male	3	2	T1N0M0	1	RT, OP
23	p85	87	Female	3	1	T2N0M0	2	OP, RT
24	p98	31	Male	3	3	T2N0M0	2	OP, RT
25	p105	63	Male	3	2	T1N0M0	1	RT, OP
26	p111	31	Female	3	2	T1N0M0	1	OP, RT
27	p119	66	Male	3	2	T2N0M0	2	OP, RT
28	p124	54	Male	3	3	T4aN2bM0	4	RT
29	p131	74	Female	3	2	T2N0M0	2	OP, RT
30	p137	71	Female	3	2	T2N0M0	2	RT, OP
31	p138	50	Male	3	2	T2N1M0	2	RT, OP

CYT, cytostatics; OP, operation; RT, radiotherapy.

* 1 = only tumor‐free sample, 2 = only tumor sample, 3 = tumor‐free and tumor samples were collected.

† 1 = tongue, 2 = lateral border of the tongue, 3 = tongue with overgrowth outside the mobile tongue.

### RNA isolation and gene expression profiling

Biopsies were fresh‐frozen in liquid nitrogen and stored at −80 °C until RNA extraction. Procedures for RNA isolation and gene expression profiling for 18 tumors, 12 tumor‐free samples, and 14 healthy controls have been previously reported and raw data were deposited in ArrayExpress accession number E‐MTAB‐4678 20. For the rest of the samples, RNA isolation was performed using AllPrep DNA/RNA/miRNA Universal Kit (Qiagen, Hilden, Germany). Quantity and purity of RNA was measured using a NanoDrop ND‐1000 spectrophotometer (ThermoScientific, Wilmington, DE, USA). RNA quality was confirmed by Agilent RNA 6000 Nano kit (Agilent 2100 Bioanalyzer, Agilent Technologies, Santa Clara, CA, USA). As reported previously, 200 ng of total RNA was processed for gene expression profiling using Illumina HumanHT‐12 v4 Expression BeadChip (Illumina Inc., San Diego, CA, USA) 20. Raw data were deposited in ArrayExpress and are available under accession number E‐MTAB‐5534. Microarray data normalization was performed using linear models and differential expression for microarray data (LIMMA) package 25, the statistical language R and extension taken from Bioconductor.

### Cell type estimation and cytolytic activity calculation

We applied the xCell method 10 to study 34 immune cell types in a total of 66 samples (21 pairs of tumor/tumor‐free samples, 8 tumor samples, 2 tumor‐free samples, and 14 control samples). Although not described in the original publication 10, xCell now also reports an ImmuneScore for each sample according to estimated levels of B cells, CD4^+^ T cells, CD8^+^ T cells, dendritic cells (DC), eosinophils, macrophages, monocytes, mast cells neutrophils, and NK cells (https://github.com/dviraran/xCell/blob/master/R/xCell.R). Granzyme A and perforin are two key cytolytic effectors that are specifically co‐expressed in cytotoxic lymphocytes 13, 14. To measure cytolytic activity in each sample according to the method of Rooney *et al* 14, microarray probe intensity data for *GZMA* and *PRF1* were extracted and the geometric mean intensity of *GZMA* and *PRF1* calculated for each sample. After that, the mean intensity value was log‐transformed and presented as cytolytic activity score.

### Confirmation of microarray data using RT‐qPCR

Levels of *GZMA* and *PRF1* mRNAs were confirmed using RT‐qPCR in 12 healthy controls and in 12 matched pairs of tumor/tumor‐free samples. RevertAid H minus first strand cDNA synthesis kit (Fermentas, ThermoScientific, Wilmington, DE, USA) was used for cDNA synthesis and qPCR was performed using an IQ5 multicolor real‐time PCR detection system with IQ SYBR Green Supermix (Bio‐Rad Laboratories, Hercules, CA, USA). Primers used were *GZMA* (forward: ATGCTATGACCCAGCCACAC, reverse: GGTTTCACATCGTCCCCCTT), *PRF1* (forward: AAGACCCACCAGGACCAGTA, reverse: TCTTGAAGTCAGGGTGCAGC), *RPL13A* (reference gene, forward: GTACGCTGTGAAGGCATCAA, reverse: GTTGGTGTTCATCCGCTTG). Primers for another reference gene *GAPDH* were ordered from Primerdesign Ltd (Southampton, UK). The primer sequences were not provided by the company.

### Statistics

Cell type composition and cytolytic activity were compared between different sample groups using nonparametric Mann–Whitney *U* test, and Spearman correlation coefficient (rho) was calculated to evaluate correlation strength. Comparisons between clinicopathological variables and cytolytic activity (low versus high) were determined by Fisher's exact test. The Kaplan–Meier method with log‐rank test was used to compare survival curves between groups. Cut‐off score for patient classification into high or low groups was chosen when showing the most significant difference. For multivariate Cox regression analysis, we considered patient age at diagnosis and TNM staging as covariates. All statistical tests were conducted in IBM SPSS Statistics 25 (IBM Corp., Armonk, NY, USA). A two‐sided *P* value <0.05 was considered significant.

## Results

### Cell type enumeration

**Figure 1:**
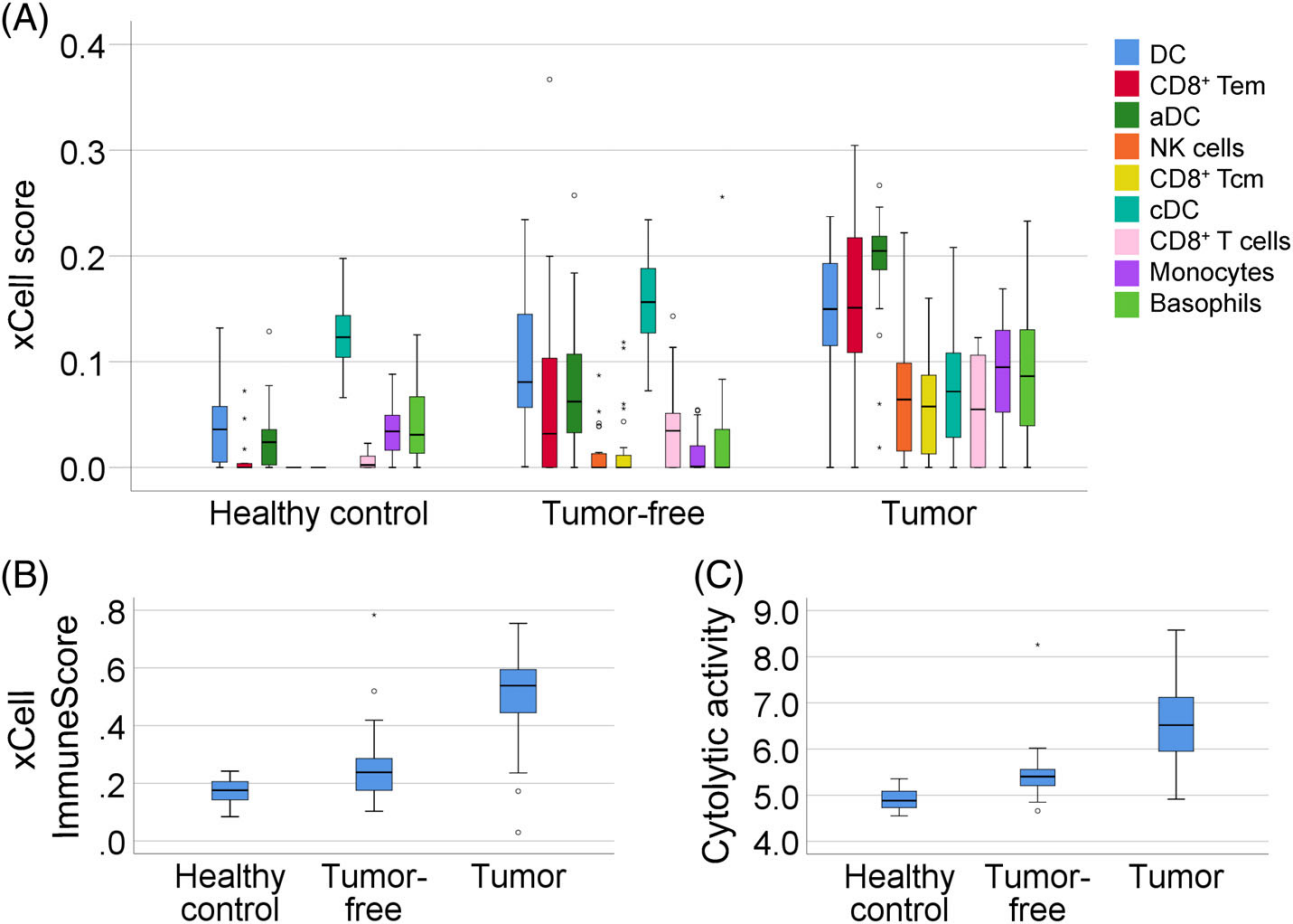
Immune features in tumor and clinically tumor‐free tongue samples from SCCOT patients compared to control tongue from healthy individuals. (A) Box‐plots of immune cell types according to xCell enumeration scores in tumor‐free samples compared to healthy controls (*p* < 0.05). Changes in the tumor samples are also shown. Small circles indicate outliers and asterisks indicate extreme outliers. (B) xCell‐derived ImmuneScores (*p* < 0.05 tumor‐free samples versus healthy control; *p* < 0.001 tumor‐free versus tumor). (C) Cytolytic activity (*p* < 0.001 tumor‐free versus healthy controls; *p* < 0.001 tumor‐free versus tumor).

Gene expression profiling data on 14 healthy controls, 23 tumor‐free and 29 tumor samples were uploaded to the xCell webtool. When comparing tumor to tumor‐free samples, there were significant alterations in all types of assessed immune cells (*p* < 0.05), except NKT cells, CD8^+^ T cells, naive B cells and plasma cells. When comparing tumor‐free samples to healthy controls, significant alterations in nine immune cell types were also seen (*p* < 0.05, Figure 1A). The most significantly elevated immune cell types in tumor‐free samples were DC, followed by CD8^+^ effector memory T cells (Tem), activated DC (aDC), NK cells, CD8^+^ central memory T cells (Tcm), conventional DC (cDC) and CD8^+^ T cells. Monocytes and basophils were significantly decreased in tumor‐free samples compared to healthy controls. The xCell calculated ImmuneScore increased from control to tumor‐free to tumor samples (Figure 1B). The ImmuneScores of all tumor and tumor‐free samples are shown in Table 2.

**Table 2:** xCell ImmuneScore and cytolytic activity score for all samples

ID	Status	Follow‐up month	Time to recurrence (month)	xCell ImmuneScore		Cytolytic activity score	
				Tumor‐free	Tumor	Tumor‐free	Tumor
p40	DWD	1		0.52		6.02	
p42	DWD	9	7	0.16		5.14	
p14	ADF	177			0.59		6.03
p24	ADF	168			0.74		6.98
p29	DWD	29	20		0.52		7.40
p68	DOD	9	6		0.44		7.16
p70	ADF	109			0.52		7.01
p82	DOD	18	12		0.60		7.12
p83	ADF	93			0.70		8.58
p92	DOD	20	6		0.73		7.54
p11	DWD	3		0.34	0.56	5.54	5.99
p35	DOD	13	10	0.14	0.03	4.85	4.97
p49	DWD	3		0.16	0.54	5.29	7.16
p51	ADF	132		0.26	0.45	5.37	5.95
p56	DOD	16	12	0.78	0.17	8.26	5.67
p58	ADF	119		0.24	0.42	5.58	6.00
p59	DOD	7		0.20	0.40	5.14	5.07
p61	DDF	81		0.22	0.54	5.45	6.59
p65	ADF	112		0.16	0.71	5.40	7.59
p73	DOD	19	11	0.24	0.58	5.13	6.45
p76	ADF	103		0.25	0.52	5.49	5.92
p79	ADF	108		0.34	0.51	5.67	6.52
p85	DOD	2	2	0.31	0.57	5.54	7.88
p98	ADF	60		0.21	0.75	4.66	6.50
p105	ADF	55		0.42	0.48	5.96	6.91
p111	ADF	51		0.19	0.26	5.27	5.40
p119	ADF	45		0.25	0.66	5.38	6.68
p124	DOD	3		0.15	0.55	5.14	5.95
p131	ADF	38		0.24	0.24	5.42	4.91
p137	ADF	36		0.10	0.56	5.34	7.02
p138	ADF	35		0.23	0.31	5.85	5.43

ADF, alive disease free; DDF, dead disease free; DOD, dead of disease; DWD, dead with disease.

### Cytolytic activity

According to our microarray data, a strong correlation between *GZMA* and *PRF1* mRNA levels was seen (Spearman correlation coefficient rho = 0.839, *p* < 0.001). Next, we calculated cytolytic activity in all 66 samples according to *GZMA* and *PRF1* levels (Table 2). Significant alterations were found not only between tumor and tumor‐free samples (*p* < 0.001), but also between tumor‐free and control samples (*p* < 0.001). Similar to the ImmuneScore, a steady increase in cytolytic activity was seen from healthy control to tumor‐free to tumor samples (Figure 1C). Spearman's correlation showed that ImmuneScore and cytolytic activity were significantly correlated (rho = 0.857, *p* < 0.001). Correlations between cytolytic activity and infiltration of a subset of immune cells were also identified. The top three correlated immune cell types were CD8^+^ Tem (rho = 0.903, *p* < 0.001), NK cells (rho = 0.842, *p* < 0.001) and activated DC (rho = 0.827, *p* < 0.001), reinforcing the reliability of cytolytic activity calculation based on *GZMA* and *PRF1* mRNA levels. To confirm the microarray data, *GZMA* and *PRF1* mRNA levels were measured using RT‐qPCR in 12 healthy controls and 12 pairs of tumor‐free and tumor samples. A significant correlation between microarray and RT‐qPCR results was seen (*GZMA*, rho = 0.897, *p* < 0.001; *PRF1*, rho = 0.691, *p* < 0.001).

### Immune features and prognosis

**Figure 2:**
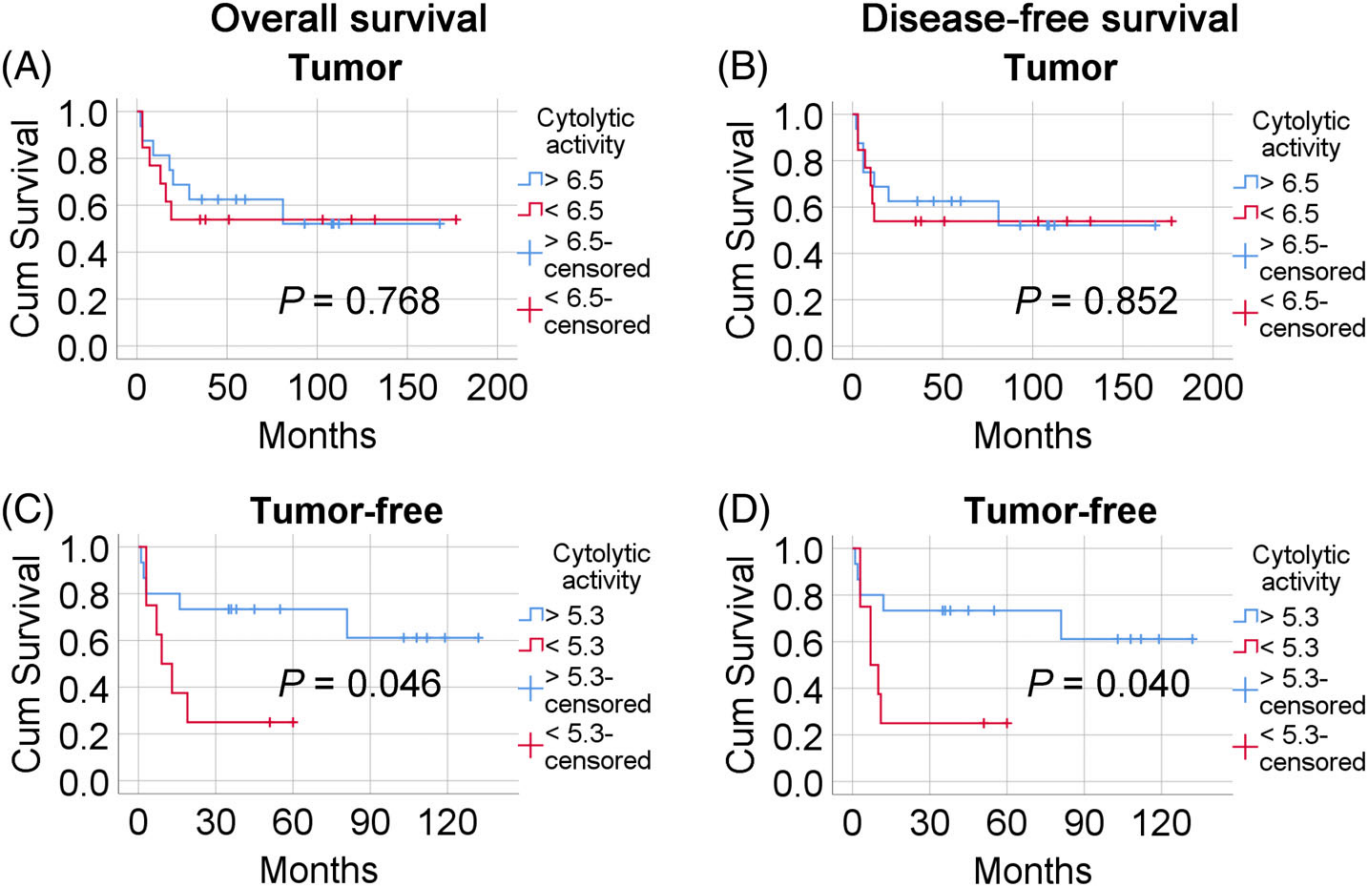
The influence of cytolytic activity in tumor and tumor‐free samples on patient survival. Kaplan–Meier curves of overall (A,C) and disease‐free (B,D) survival are shown. Blue lines represent patients with high cytolytic activity and red lines patients with low cytolytic activity. (A,B) Tumor samples (C,D) Tumor‐free samples.

As tumor‐related immune features have been shown to be prognostic across several tumor types, we investigated the effect of immune infiltration on SCCOT prognosis. Overall survival was defined as the time from date of completion of first‐line treatment to death, and disease free survival as the time from date of completion of first‐line treatment to date of first recurrence or of death without recurrence. Patients were divided into high or low score groups according to immune cell composition, ImmuneScore or cytolytic activity in their tumor samples. Kaplan–Meier analysis showed no significant difference in clinical outcome of patients with high or low scores (Figure 2A,B). Next, we divided patients into high or low score groups according to immune cell composition, ImmuneScore or cytolytic activity in their tumor‐free samples. Cytolytic activity correlated with patient survival, whereas there were no associations with immune cell composition or ImmuneScore. As shown in Figure 2C, patients with high cytolytic activity in their tumor‐free tissue (*n* = 15) had improved overall survival compared to patients with low cytolytic activity in their tumor‐free samples (*n* = 8, *p* = 0.046). A correlation between cytolytic activity and disease‐free survival was also seen (*p* = 0.040, Figure 2D). There was no significant difference in age, sex, tumor size and stage between high or low score groups (Table 3); however, within the survival data for high cytolytic activity patients, we found that the three patients who had died within 3 months were all 78 years or older (patient numbers 1, 11, and 23). In subsequent multivariate Cox regression analysis adjusted for tumor stage and patient age, cytolytic activity in tumor‐free samples remained an independent prognostic factor for disease‐free survival (hazard ratio = 4.20, 95% CI = 1.09–16.20, *p* = 0.037).

**Table 3:** Associations between clinicopathological variables and cytolytic activity

Variable	Low cytolytic activity in tumor‐free samples (*n* = 8)	High cytolytic activity in tumor‐free samples (*n* = 15)	*P* value
Age (years, mean)	51	67	0.057 (Mann–Whitney *U* test)
Sex (female/male)	5/3	6/9	0.400 (Fisher's exact test)
Tumor size (1,2/4)	5/3	12/3	0.621 (Fisher's exact test)
Tumor Stage (I, II/III, IV)	5/3	10/5	1.000 (Fisher's exact test)

## Discussion

Multiple studies have shown that infiltration of immune cells into the tumor microenvironment is a prognostic factor in cancer. Recent studies focusing on tumor immune cytolytic activity also demonstrated that transcript levels of two key cytolytic effectors, *GZMA* and *PRF1*, correlate with patient survival 14, 26. In this study, using transcriptome profiling data, we estimated immune cell composition and cytolytic activity in SCCOT, clinically tumor‐free tongue tissue from SCCOT patients and control tongue tissue from healthy individuals. Correlations between immune infiltration, cytolytic activity and patient survival were also investigated. As expected, significantly altered immune cell composition was seen in tumors compared to tumor‐free samples. However, we also found elevated infiltration of DC, CD8^+^ T cells and NK cells in tumor‐free tongue compared to control samples from healthy individuals, whereas infiltration of monocytes and basophils showed a decrease within the tumor‐free samples. The overall ImmuneScore was also higher in tumor‐free samples compared to healthy controls. Importantly, we also found increased cytolytic activity in tumor‐free samples compared to healthy controls. Therefore, similar to a recent report that several immune cell types are highly enriched in normal tissue adjacent to tumor compared with healthy tissue in eight different tissue types (bladder, breast, colon, liver, lung, prostate, thyroid, and uterus) 11, we demonstrate the presence of an expanded field of immunoreactivity in clinically tumor‐free tongue tissue in SCCOT patients. It should be noted that unlike other sites within the head and neck region, such as oropharynx and nasopharynx, there appears to be no role for viral infections (either human papillomavirus or Epstein–Barr virus) in SCCOT 23, 24. Thus, viral influences are unlikely to account for any variations in the immunoreactivity.

We also found that patients with high cytolytic activity in tumor‐free tongue had improved survival compared with patients with low cytolytic activity. Cytolytic activity in tumors has been shown to correlate with mutation load and number of predicted neoantigens 14, 26. Oral SCC, including SCCOT, is a paradigm of Slaughter's concept of ‘field cancerization’ 27, in which tumors are thought to arise from an expanded pool of genetically altered pre‐neoplastic cells 28, 29. This concept has been modified to include exposure of the tissue microenvironment to damaging/mutagenic agents, termed ‘etiologic field effects’ 30, 31. The recently identified changes in gene expression profiles in clinically tumor‐free tongue in patients with SCCOT compared to healthy controls provide definitive evidence for field effects in this disease 20. Therefore, SCCOT patients with high cytolytic activity in the tumor‐free parts of the tongue could be indicative of immunogenicity to cells with high mutation burden in the cancer field and/or immune responses due to etiologic field effects. It has been reported that overall gene expression profiles of histologically normal oral mucosa are useful in identifying markers for clinical outcome and recurrence in patients with oral SCC 32, 33. Here, we found that cytolytic activity in the tumor‐free tongue in patients with SCCOT provides prognostic information. In contrast, levels of immune infiltration or degree of cytolytic activity within the tumor is not predictive for patient survival. Thus, measuring cytolytic activity in tumor‐free samples contralateral to the tumor could be an effective approach for evaluating prognosis in patients with SCCOT.

Unlike ‘ImmuneScore’, a methodology based on immunohistochemistry and derived from the density and location of two lymphocyte populations 7, the xCell reported an ‘ImmuneScore’ derived from estimated levels of B cells, CD4^+^, and CD8^+^ T cells, DC, eosinophils, macrophages, monocytes, mast cells, neutrophils and NK cells. As the functional plasticity of immune cells is not fully understood and information on cell location is lacking, the value of bulk gene expression data based ‘ImmuneScore’ in clinical practice is limited.

There are two potential limitations to our study. First, the number of samples analyzed is relatively small, due to the difficulties in obtaining sufficient control and tumor‐free samples. Second, sample size excludes the ability for immunohistochemical confirmation of the data. Nonetheless, our novel analyses provide a useful approach to investigate immune activity in clinical samples and identify significant associations with patient prognosis for further investigation.

In conclusion, elevated cytolytic activity was seen in tumor‐free tissue from SCCOT patients, where it was found to be an independent prognostic factor for disease‐free survival. Whilst the reason(s) for this association are at present unclear, integrating immunogenomic data from tumor‐free and tumor samples to characterize the immune microenvironment in SCCOT could help predict clinical outcome for patients with SCCOT.

## Author contributions statement

XG designed and performed experiments, analyzed data and wrote the manuscript. LB designed and performed experiments, analyzed data and wrote the manuscript. PJC analyzed data and wrote the manuscript. RF analyzed data and wrote the manuscript. LW analyzed data. TW and LNS provided medical materials. NS analyzed data. KN supervised the project and wrote the manuscript. All authors commented on the manuscript.

## References

[1] Binnewies M , Roberts EW , Kersten K , *et al* Understanding the tumor immune microenvironment (TIME) for effective therapy. Nat Med 2018; 24: 541–550. 29686425 10.1038/s41591-018-0014-x PMC5998822

[2] Baumeister SH , Freeman GJ , Dranoff G , *et al* Coinhibitory pathways in immunotherapy for cancer. Annu Rev Immunol 2016; 34: 539–573. 26927206 10.1146/annurev-immunol-032414-112049

[3] Hackl H , Charoentong P , Finotello F , *et al* Computational genomics tools for dissecting tumour‐immune cell interactions. Nat Rev Genet 2016; 17: 441–458. 27376489 10.1038/nrg.2016.67

[4] Charoentong P , Finotello F , Angelova M , *et al* Pan‐cancer immunogenomic analyses reveal genotype‐immunophenotype relationships and predictors of response to checkpoint blockade. Cell Rep 2017; 18: 248–262. 28052254 10.1016/j.celrep.2016.12.019

[5] Thorsson V , Gibbs DL , Brown SD , *et al* The immune landscape of cancer. Immunity 2018; 48: 812–830 e814. 29628290 10.1016/j.immuni.2018.03.023 PMC5982584

[6] Gentles AJ , Newman AM , Liu CL , *et al* The prognostic landscape of genes and infiltrating immune cells across human cancers. Nat Med 2015; 21: 938–945. 26193342 10.1038/nm.3909 PMC4852857

[7] Galon J , Mlecnik B , Bindea G , *et al* Towards the introduction of the 'Immunoscore' in the classification of malignant tumours. J Pathol 2014; 232: 199–209. 24122236 10.1002/path.4287 PMC4255306

[8] Pages F , Mlecnik B , Marliot F , *et al* International validation of the consensus Immunoscore for the classification of colon cancer: a prognostic and accuracy study. Lancet 2018; 391: 2128–2139. 29754777 10.1016/S0140-6736(18)30789-X

[9] Finotello F , Trajanoski Z . Quantifying tumor‐infiltrating immune cells from transcriptomics data. Cancer Immunol Immunother 2018; 67: 1031–1040. 29541787 10.1007/s00262-018-2150-z PMC6006237

[10] Aran D , Hu Z , Butte AJ . xCell: digitally portraying the tissue cellular heterogeneity landscape. Genome Biol 2017; 18: 220. 29141660 10.1186/s13059-017-1349-1 PMC5688663

[11] Aran D , Camarda R , Odegaard J , *et al* Comprehensive analysis of normal adjacent to tumor transcriptomes. Nat Commun 2017; 8: 1077. 29057876 10.1038/s41467-017-01027-z PMC5651823

[12] Martinez‐Lostao L , Anel A , Pardo J . How do cytotoxic lymphocytes kill cancer cells? Clin Cancer Res 2015; 21: 5047–5056. 26567364 10.1158/1078-0432.CCR-15-0685

[13] Voskoboinik I , Whisstock JC , Trapani JA . Perforin and granzymes: function, dysfunction and human pathology. Nat Rev Immunol 2015; 15: 388–400. 25998963 10.1038/nri3839

[14] Rooney MS , Shukla SA , Wu CJ , *et al* Molecular and genetic properties of tumors associated with local immune cytolytic activity. Cell 2015; 160: 48–61. 25594174 10.1016/j.cell.2014.12.033 PMC4856474

[15] Bray F , Ferlay J , Soerjomataram I , *et al* Global cancer statistics 2018: GLOBOCAN estimates of incidence and mortality worldwide for 36 cancers in 185 countries. CA Cancer J Clin 2018; 68: 394–424. 30207593 10.3322/caac.21492

[16] Leemans CR , Snijders PJF , Brakenhoff RH . The molecular landscape of head and neck cancer. Nat Rev Cancer 2018; 18: 269–282. 29497144 10.1038/nrc.2018.11

[17] Solomon B , Young RJ , Rischin D . Head and neck squamous cell carcinoma: genomics and emerging biomarkers for immunomodulatory cancer treatments. Semin Cancer Biol 2018; 52: 228–240. 29355614 10.1016/j.semcancer.2018.01.008

[18] Boldrup L , Coates PJ , Wahlgren M , *et al* Subsite‐based alterations in miR‐21, miR‐125b, and miR‐203 in squamous cell carcinoma of the oral cavity and correlation to important target proteins. J Carcinog 2012; 11: 18. 23230394 10.4103/1477-3163.104007 PMC3515918

[19] Cancer Genome Atlas N . Comprehensive genomic characterization of head and neck squamous cell carcinomas. Nature 2015; 517: 576–582. 25631445 10.1038/nature14129 PMC4311405

[20] Boldrup L , Gu X , Coates PJ , *et al* Gene expression changes in tumor free tongue tissue adjacent to tongue squamous cell carcinoma. Oncotarget 2017; 8: 19389–19402. 28038473 10.18632/oncotarget.14288 PMC5386692

[21] Gu X , Boldrup L , Coates PJ , *et al* Epigenetic regulation of OAS2 shows disease‐specific DNA methylation profiles at individual CpG sites. Sci Rep 2016; 6: 32579. 27572959 10.1038/srep32579 PMC5004144

[22] Lundqvist L , Stenlund H , Laurell G , *et al* The importance of stromal inflammation in squamous cell carcinoma of the tongue. J Oral Pathol Med 2012; 41: 379–383. 22084865 10.1111/j.1600-0714.2011.01107.x

[23] Sgaramella N , Coates PJ , Strindlund K , *et al* Expression of p16 in squamous cell carcinoma of the mobile tongue is independent of HPV infection despite presence of the HPV‐receptor syndecan‐1. Br J Cancer 2015; 113: 321–326. 26057450 10.1038/bjc.2015.207 PMC4506391

[24] Wilms T , Khan G , Coates PJ , *et al* No evidence for the presence of Epstein‐Barr virus in squamous cell carcinoma of the mobile tongue. PLoS One 2017; 12: e0184201. 28926591 10.1371/journal.pone.0184201 PMC5604943

[25] Shi W , Oshlack A , Smyth GK . Optimizing the noise versus bias trade‐off for Illumina whole genome expression BeadChips. Nucleic Acids Res 2010; 38: e204. 20929874 10.1093/nar/gkq871 PMC3001098

[26] Narayanan S , Kawaguchi T , Yan L , *et al* Cytolytic activity score to assess anticancer immunity in colorectal Cancer. Ann Surg Oncol 2018; 25: 2323–2331. 29770915 10.1245/s10434-018-6506-6 PMC6237091

[27] Slaughter DP , Southwick HW , Smejkal W . Field cancerization in oral stratified squamous epithelium; clinical implications of multicentric origin. Cancer 1953; 6: 963–968. 13094644 10.1002/1097-0142(195309)6:5<963::aid-cncr2820060515>3.0.co;2-q

[28] Leemans CR , Braakhuis BJ , Brakenhoff RH . The molecular biology of head and neck cancer. Nat Rev Cancer 2011; 11: 9–22. 21160525 10.1038/nrc2982

[29] Curtius K , Wright NA , Graham TA . An evolutionary perspective on field cancerization. Nat Rev Cancer 2018; 18: 19–32. 29217838 10.1038/nrc.2017.102

[30] Lochhead P , Chan AT , Nishihara R , *et al* Etiologic field effect: reappraisal of the field effect concept in cancer predisposition and progression. Mod Pathol 2015; 28: 14–29. 24925058 10.1038/modpathol.2014.81 PMC4265316

[31] Dotto GP . Multifocal epithelial tumors and field cancerization: stroma as a primary determinant. J Clin Invest 2014; 124: 1446–1453. 24691479 10.1172/JCI72589 PMC3973113

[32] Lohavanichbutr P , Houck J , Doody DR , *et al* Gene expression in uninvolved oral mucosa of OSCC patients facilitates identification of markers predictive of OSCC outcomes. PLoS One 2012; 7: e46575. 23029552 10.1371/journal.pone.0046575 PMC3460916

[33] Reis PP , Waldron L , Perez‐Ordonez B , *et al* A gene signature in histologically normal surgical margins is predictive of oral carcinoma recurrence. BMC Cancer 2011; 11: 437. 21989116 10.1186/1471-2407-11-437 PMC3198722

